# Supplementary material for: Modified gemcitabine, S-1, and leucovorin combination for patients with newly diagnosed locally advanced or metastatic pancreatic adenocarcinoma: A multi-center retrospective study in Taiwan
Source: PLoS One. 2020 Dec 29;15(12):e0244487. doi: 10.1371/journal.pone.0244487 (PMC7771868; doi:10.1371/journal.pone.0244487)
Supplement: Attachment — Submitted filename: GSL in PC Revised Letter-PLOS ONE-20201206.doc [file pone.0244487.s002.doc]

Response to Editor

Ref.: PONE-D-20-24681

Modified Gemcitabine, S-1, and Leucovorin Combination for Patients with Newly Diagnosed Advanced or Metastatic Pancreatic Adenocarcinoma: A Multi-center Retrospective Study in Taiwan

Dear Editor-in-Chief,

We would like to thank the reviewers for their valuable and constructive comments, and have revised the manuscript accordingly. The revised paragraphs with tract changes are highlighted in red, and the point-by-point responses to the reviewer’s comments are summarized as follows.

Editor comments:

1. Please ensure that your manuscript meets PLOS ONE's style requirements, including those for file naming. The PLOS ONE style templates can be found at https://journals.plos.org/plosone/s/file?id=wjVg/PLOSOne_formatting_sample_main_body.pdf and https://journals.plos.org/plosone/s/file?id=ba62/PLOSOne_formatting_sample_title_authors_affiliations.pdf

🡪 The whole manuscript has been revised to meet the PLOS ONE’s style requirements.

2. Thank you for including your ethics statement: 'The study was approved by the Local Ethics Committee (CMUH107-REC1-140) with a waiver of informed consent.' (a) Please amend your current ethics statement to include the full name of the ethics committee/institutional review board(s) that approved your specific study. (b) Once you have amended this/these statement(s) in the Methods section of the manuscript, please add the same text to the “Ethics Statement” field of the submission form (via “Edit Submission”).

🡪 The full name of the ethics committee has been added in the paragraph of “Materials and Methods” (line 133) and the “Ethics Statement” field online.

3. In the ethics statement in the manuscript and in the online submission form, please provide additional information about the patient records used in your retrospective study, including: a) whether all data were fully anonymized before you accessed them and b) the date range (month and year) during which patients' medical records were accessed.

🡪 Per suggestion, the relevant statement and date range for data collection are added in “Materials and Methods” (line 136-137).

4. To comply with PLOS ONE submission guidelines, in your Methods section, please provide additional information regarding your statistical analyses. For more information on PLOS ONE's expectations for statistical reporting, please see https://journals.plos.org/plosone/s/submission-guidelines.#loc-statistical-reporting.

🡪 We have revised the relevant statement for statistical analysis (line 170-174).

5. Your ethics statement should only appear in the Methods section of your manuscript. If your ethics statement is written in any section besides the Methods, please delete it from any other section.

🡪We have confirmed that the ethics statement is listed in only the Materials and Methods section.

Reviewers' comments:
Reviewer #1:

Q1: In the title please modify Advanced in Locally Advanced.

🡪 It has been revised accordingly (line 3).

Q2: In the paragraph "Introduction": on line 77 please correct trial to trials; on line 82 please specify that the considerable toxicities are related to FOLFIRINOX; on line 89 however should be substituted with despite of modified schedule; on line 113 please specify that the reference [8] is referred to gastric and not pancreatic cancer.

🡪 Thank you for suggestions. All points are revised accordingly (line 80, 85, 93, and 117).

Q3:In the paragraph “Discussion” authors should better explain the reasons why gemcitabine was given at a 20% lower dose (800 mg instead of 1000 mg/m2) and with a modified schedule (on day 1 every 2 weeks instead of days 1 and 8 every 3 weeks). Indeed similar modifications of dosage and schedule could have affected safety and toxicity results; authors should mention and argue the point.

🡪 We thank you the great question. The rationale and background of using biweekly gemcitabine at 800 mg/m2 are added in Discussion (line 254-261, 276-283).

Reviewer #2:

Q1: This is a retrospective study of a chemotherapy regimen GSL for patients with locally advanced and/or metastatic pancreas cancer. There are only 49 patients in the study. The toxicity was manageable so the chemotherapy was reasonably well tolerated. However, the efficacy was minimal as the median survival was 10 months and the median progression-free survival was 8 months. The regimen has minimal efficacy.

🡪 Thank you for the question. The major point of this retrospective study is to show the relatively lower toxicities of GSL regimen compared with previous standard regimens without compromising the activity for patients with pancreatic cancer. The median progression-free survival and overall survival time was 6.94 and 11.53 months, and 5.7 and 10.1 months in our study and in the phase III GEST study (GS arm), respectively. Because of the modest activity and safety profiles, a prospective trial evaluating the same regimen in elderly patients with locally advanced or metastatic pancreatic adenocarcinoma is currently ongoing in Taiwan.

Reviewer #3:

Q1: Results are limited to the description of survival parameters, objective response, toxicity. In order to better characterize the response (efficacy/toxicity) of this setting of patients in respect to the study combination, a proper statistical analysis (univariate and multivariate analysis) of relationships between efficacy (survival parameters - PFS and OS; objective response) or toxicity and clinical/pathological characteristics included in Table 1 should be performed.

🡪 Thank you for great suggestion. The univariate and multivariate analyses of risk factors for progression-free survival and overall survival have been performed and been shown in new Table 3 and 4.

Q2: The statistical analysis should also include as variables to be considered the administered cycles and dose reductions (i.e. by subdividing patients according to at least two groups) and second line treatments. Please, add in table 1, accordingly, the mean number of administered cycles (and range), dose reductions and second line treatments.

🡪The information of administration cycles, dose reduction and second line therapy are added in Table 1 and content (line 201-203). We also include the administration cycle, dose reduction and second line therapy as parameters in the univariate and multivariate analyses for progression-free survival and overall survival (Table 3 and 4).

Q3: English language should be revised by a mother tongue person.

🡪 Thank you for your suggestion. We have revised and polished the manuscript with the help of an English editing company Editage for its linguistic assistance. The proof of the service provided by Editage is attached here for your reference.

Q4: Please, include in Table 3 also the absolute numbers of patients and not only percentages.

🡪 The numbers and percentages of patients have been added in new Table 5.

Thank you for your very valuable feedback and critique.

Yours sincerely,

Li-Yuan Bai, M.D., Ph.D.

Division of Hematology and Oncology, Department of Medicine, China Medical University Hospital, 2, Yude road, Taichung 40402, Taiwan.

Phone: +886-4-22052121 ext 5051

Fax: +886-4-22337675

E-mail: lybai6@gmail.com

.
